# Screening a Natural Product-Inspired Library for Anti-*Phytophthora* Activities

**DOI:** 10.3390/molecules26071819

**Published:** 2021-03-24

**Authors:** Scott A. Lawrence, Hannah F. Robinson, Daniel P. Furkert, Margaret A. Brimble, Monica L. Gerth

**Affiliations:** 1 Department of Microbiology and Immunology, University of Otago, Dunedin 9054, New Zealand; Scott.Lawrence@otago.ac.nz; 2 School of Biological Sciences, Victoria University of Wellington, Wellington 6140, New Zealand; hannah.robinson@vuw.ac.nz; 3 School of Chemical Sciences, University of Auckland, Auckland 1142, New Zealand; d.furkert@auckland.ac.nz (D.P.F.); m.brimble@auckland.ac.nz (M.A.B.)

**Keywords:** natural products, antimicrobial, *Phytophthora agathidicida*, *Phytophthora cinnamomi*

## Abstract

*Phytophthora* is a genus of microorganisms that cause devastating dieback and root-rot diseases in thousands of plant hosts worldwide. The economic impact of *Phytophthora* diseases on crops and native ecosystems is estimated to be billions of dollars per annum. These invasive pathogens are extremely difficult to control using existing chemical means, and the effectiveness of the few treatments available is being jeopardized by increasing rates of resistance. There is an urgent need to identify new chemical treatments that are effective against *Phytophthora* diseases. Natural products have long been regarded as “Nature’s medicine chest”, providing invaluable leads for developing front-line drugs and agrochemical agents. Here, we have screened a natural product-inspired library of 328 chemicals against two key *Phytophthora* species: *Phytophthora cinnamomi* and *Phytophthora agathidicida*. The library was initially screened for inhibition of zoospore germination. From these screens, we identified twenty-one hits that inhibited germination of one or both species. These hits were further tested in mycelial growth inhibition studies to determine their half-maximal inhibitory concentrations (IC_50_s). Four compounds had IC_50_ values of approximately 10 µM or less, and our best hit had IC_50_s of approximately 3 µM against both *Phytophthora* species tested. Overall, these hits may serve as promising leads for the development of new anti-*Phytophthora* agrochemicals

## 1. Introduction

*Phytophthora* (from the Greek for “plant-destroyers”) is a genus of plant pathogens in the class Oomycota. Oomycetes, or water molds, are eukaryotic microorganisms that superficially resemble fungi but are phylogenetically closer to algae [1]. Dieback and root rot diseases caused by *Phytophthora* species are notoriously difficult to control or eradicate. *Phytophthora* lack many common fungicide targets, such as the ergosterol biosynthesis pathway and chitin-based cell walls [2,3]. Compared to true fungi, *Phytophthora* also have a more complex lifecycle that includes not only fungus-like mycelia but also short-lived, motile zoospores and long-lived oospores and/or chlamydospores.

Globally, *Phytophthora* diseases cost the horticultural industry billions of dollars each year. One disease alone, potato late blight (caused by *P. infestans*), causes annual revenue losses of hundreds of millions of dollars in the USA through treatment costs and crop loss [4]. The effects of *Phytophthora* on natural ecosystems are equally devastating; sudden oak death caused by *P. ramorum* and root rot caused by *P. cinnamomi* are just two examples of *Phytophthora* diseases causing widespread destruction in the forests of the USA and Australia, respectively [5,6]. Given the widespread economic and ecological damage these organisms cause, there is a pressing need to develop new preventative and/or curative options for *Phytophthora* diseases.

Natural products have long been regarded as “Nature’s medicine chest”, providing invaluable scaffolds for developing front-line drugs and agrochemical agents. The chemical structures of natural products have evolved over several millennia for specific biochemical purposes, and their molecular frameworks can be considered “privileged scaffolds” [7]. Examples of natural products with promising anti-oomycete activity include the glucosinolates (and their hydrolysis products, isothiocyanates) present in the Brassicaceae plant family, which recent studies have shown are effective against different lifecycle stages of several *Phytophthora* species [8,9,10]. Similarly, metabolites from fungal extracts have also been shown to have anti-*Phytophthora* activities [11]. In this study, we have screened a natural product-inspired library of 328 chemicals against two key *Phytophthora* species: *Phytophthora cinnamomi* and *Phytophthora agathidicida*.

*Phytophthora cinnamomi* is a broad host range pathogen that infects ~5000 species of plants, devasting natural ecosystems, agriculture, forestry and horticulture worldwide [6]. *Phytophthora agathidicida* is the causative agent of kauri dieback, a disease afflicting the kauri (*Agathis australis*) forests of northern New Zealand. Kauri are one of the largest and longest-living tree species in the world, and these trees are both culturally and ecologically significant to New Zealand [12].

At present, one of the most effective treatments for both *Phytophthora* species is potassium phosphite [12,13,14,15], which functions both as an antimicrobial and as an inducer of host defence mechanisms [16,17,18]. While phosphite is relatively effective and has low environmental toxicity, it does not eradicate the pathogens, meaning ongoing treatment is required [14]. Further, there is evidence of resistance developing in some *Phytophthora* species [19,20,21]. Given the above limitations, the scarcity of alternative control options and the environmental concerns surrounding the use of existing fungicides [22], there is an urgent need to discover and develop new anti-*Phytophthora* treatments. Here, we describe the results of screening 328 nature-inspired products, intermediates and organocatalysts for activity against *P. agathidicida* and *P. cinnamomi*. 

## 2. Results and Discussion

**Figure 1:**
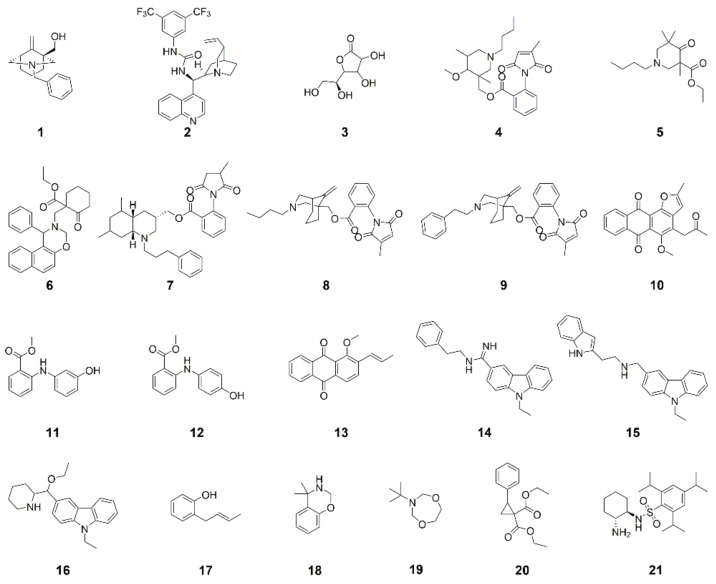
Hits from the initial screening of a library of 328 compounds for inhibition of *P. agathidicida* and/or *P. cinnamomi* zoospore germination.

In the present study, we used a microtiter plate-based assay to screen a library of 328 natural product-inspired compounds (Supplementary Table S1) for anti-*Phytophthora* activity. Our initial screen focused on the inhibition of zoospore germination. This initial screen revealed twenty-one compounds (Figure 1) that prevented zoospore germination for at least one of the *Phytophthora* species tested. 

Of these twenty-one hits, fifteen inhibited both species (**2**–**5**, **8**, **10**–**15**, **17**, **19**–**21**), three inhibited *P. cinnamomi* only (**6**, **7**, **18**) and three inhibited *P. agathidicida* only (**1**, **9**, **16**) only. 

The twenty-one compounds from the initial screen were next assayed for inhibition of mycelial growth at a range of concentrations to determine their IC_50_s. For compounds that were active against both species, IC_50_ values for mycelial inhibition were generally very similar between the species (Table 1). 

Four of the compounds (**2**, **15**, **20** and **21**) showed particularly strong mycelial growth inhibition, with IC_50_ values of ~10 µM or less for at least one of the species tested. Overall, the most potent compound was **20**, with IC_50_ values of ~3 µM for both species. These results compare favourably with existing oomycides such as copper sulfate and fosetyl-aluminium (*P. cinnamomi* IC_50_ values of ~30 and 150 µM, respectively) [23,24]. The compounds also show stronger inhibition than several other natural products with anti-*Phytophthora* activity. Examples targeting *P. cinnamomi* include a plant-derived triterpenoid (76% growth inhibition at 200 µM) [25]; *Phlomis purpurea* root extract (85.5% inhibition at 10 mg/mL) [26]; flufuran and derivative compounds (10–100% inhibition at ~1.4 mM) [27], *Magnolia vovidessi* extracts (~30–70% inhibition at 2 mg/mL) [28] and pomegranate extract (~40% inhibition at 10 mg/mL) [29]. 

Though there were no striking structural similarities between our top four compounds, there were a number of recurring moieties amongst the other hits. These promising scaffolds for further lead development are discussed below. 

The structures of **4**, **7**, **8** and **9** all contain an *N*-substituted anthranilate ester and are based on the diterpene alkaloid methyllycaconitine (MLA), which is produced by plants of the *Delphinium* and *Aconitum* genera [30,31,32]. MLA is known to be a nicotinic acetylcholine receptor antagonist in mammals and insects, and structure-activity studies of MLA have demonstrated the importance of the *N*-substituted anthranilate ester moiety and *N*-ethyl tertiary amine for its activity [33,34]. Therefore, several research groups have explored the incorporation of these functional groups into simpler, MLA-related compounds, such as **4**, **7**, **8** and **9**, for use as pesticides or pharmacological tools [30,35,36,37,38,39,40]. For **4**, **7**, **8** and **9**, the *N*-substitution of the anthranilate ester is a methylsuccinimide or methylmaleimide group. Nicotinic acetylcholine receptors are absent in oomycetes [41], so the anti-*Phytophthora* activity of **4**, **7**, **8** and **9** observed in this work is not likely to be due to nAChR antagonism. Other maleimide-containing compounds have been reported to have antifungal and/or anti-*Phytophthora* activities [42,43]; however, the mode-of-action of these compounds is currently unknown. Interestingly, **7** and **9** displayed species-specific activities with **7** being active against only *P. cinnamomi* and **9** against only *P. agathidicida*, while **4** and **8** were active against both species. The only structural difference between **8** and **9** is in the *N*-substitution of the tertiary amine: **4** and **8** contain an *N*-butyl group, but **7** and **9** contain an *N*-propylphenyl and *N*-ethylphenyl group, respectively. Therefore, this is a possible moiety of interest in future structure-activity studies.

As with **4**, **7**, **8** and **9**, compounds **11** and **12** also contain an *N*-substituted anthranilate ester. Both **11** and **12** are intermediates in the synthesis of inhibitors of *Mycobacterium tuberculosis* (*Mtb*) anthranilate phosphoribosyltransferase (AnPRT), which catalyzes a key step in the tryptophan biosynthesis pathway. Tryptophan biosynthesis is essential for *Mtb* pathogenesis and, therefore, is a target for the development of anti-*Mtb* drugs [44,45,46,47,48]. Oomycetes, including *Phytophthora*, also use an AnPRT for tryptophan biosynthesis [49]. In the present study, ten additional compounds structurally similar to **11** and **12** were tested in mycelial IC_50_ assays against *P. cinnamomi* and *P. agathidicida* to look for evidence of structure-activity relationships. Activity varied among these compounds and was often substantially lower than **11** and **12** (Supplementary Table S2), but four of the compounds were, to a degree, active against both species. Substitution of both phenyl groups at the position *ortho* to the amine with a methyl ester and substitution of the hydroxy group resulted in decreased activity. 

Multiple hits contain an anthraquinone scaffold (i.e., **10** and **13**). Anthraquinones are anthracene (three fused benzene rings)-derived structures, but the central ring contains two carbonyl groups, thus providing the quinone substructure. In nature, anthraquinones and their derivatives are produced as secondary metabolites by plants, lichens, insects and higher filamentous fungi. Naturally occurring anthraquinones extracted from plants have long been used in foods, cosmetics and traditional Chinese medicine [50]. Anthraquinones are also increasingly being explored as privileged scaffolds for pharmaceutical development due to their antimicrobial, anti-cancer and anti-thrombotic activities [51,52,53]. Various anthraquinones produced by plants have also been shown to have anti-*Phytophthora* activities [54,55,56,57]. However, this study is the first to report the anti-*Phytophthora* activities of **10** and **13**.

The structures of hits **14**, **15** and **16** all share a carbazole scaffold. Carbazoles are tricyclic aromatic compounds containing two benzene rings fused to either side of a pyrrole, and the majority of carbazole alkaloids have been isolated from plants of the *Murraya*, *Glycosmis* and *Clausena* genera and Rutaceae family [58]. These compounds are reported to have antimicrobial, insecticidal and antiprotozoal activity [59,60]. For example, a series of carbazole ellipticinium derivatives have notable inhibitory activity against *P. infestans* [61,62]. In the present study, a further fourteen compounds with structural similarity to **15** were tested in the mycelial IC_50_ assays against *P. cinnamomi* and *P. agathidicida*. Most were found to have activity, albeit lower than **15**, against both species, suggesting a weak structure-activity relationship in the mechanism of action. Alterations to the identity (e.g., containing pyridine or piperidine), branching and position of the pendant groups of the ethylcarbazole resulted in decreased activity (Supplementary Table S3).

Overall, the hits identified herein represent promising leads and/or chemical scaffolds for further development of oomycides. Of particular interest are the six compounds that were active against only one of the *Phytophthora* species tested (**1**, **6**, **7**, **9**, **16**, **18**). This specificity suggests targeted inhibition of one or more proteins, which is desirable for the development of species-specific antimicrobials. While promising, any off-target activity would need to be confirmed via phytotoxicity testing, antimicrobial assays and/or community sequencing prior to the implementation of potential oomycides in the field. Selective toxicity will be important for the control of *P. agathidicida* in particular, given the ecological significance of kauri trees and their surrounding biota. Future work could include medicinal chemistry studies to further explore the structure-activity relationships of these hits to improve potency and solubility (which relates to systemic movement in the plant) and could also include explorations into the mode of action of these compounds. 

## 3. Materials and Methods

### 3.1. Compound Library

A set of 328 compounds with diverse chemical structures were selected from a larger 3500 compound library synthesized by the Brimble Lab at the University of Auckland, New Zealand. The full list of compounds screened can be found in Supplementary Table S1. The compounds in this set were produced during extensive synthetic work towards a range of diverse bioactive natural products and related scaffolds [31,32,39,40,47,60,63,64,65,66,67,68,69,70]. Additional information on individual compounds is available upon request. For anti-*Phytophthora* screening, compounds were resuspended in DMSO at a concentration of 10 mM and diluted to 100 µM with sterile Milli-Q water for use in assays. 

### 3.2. Phytophthora Isolates and Culture Conditions

*P. agathidicida* isolate NZFS 3770 and *P. cinnamomi* isolate NZFS 3910 (both provided by Scion, Rotorua, New Zealand) were routinely cultured at 22 °C in darkness on clarified 20% V8 juice agar (cV8-agar) as described previously [71].

For zoospore production, 10 agar plugs (6 mm diameter) were removed from the edge of actively growing mycelia and then transferred to a petri dish containing 15 mL of a 1:10 dilution of 20% cV8 broth (*P. cinnamomi*) or carrot broth (*P. agathidicida*). These were grown for ~30 h at 25 °C. Broth was then removed and replaced with 15 mL of Chen-Zentmyer salt solution (for *P. cinnamomi*) or 5% (*w*/*v*) sterile soil extract (for *P. agathidicida*). Dishes were incubated at room temperature for 45 min. Then, the solutions were removed and replaced with fresh salt solution/soil extract. This was repeated after another 45 min, and the dishes were then incubated overnight at room temperature under light. The following morning, zoospore release was induced by removing the liquid and washing each dish three times with sterile Milli-Q water that had been cooled to 4 °C. Each wash was for 20 min, with the first two at room temperature and the final wash at 4 °C. Wash volumes were 15 mL per dish for the first two washes and 10 mL for the final wash. Following the final wash, dishes were returned to room temperature for 30–90 min until sufficient number of zoospores had been released. Zoospore densities were approximately 1500 per mL.

### 3.3. Inhibition Assays

Initial screening of the 328 compounds against zoospore germination was done in 96-well plates, with each well containing 100 µL of 4% cV8 broth amended with one of the compounds to a concentration of 200 µM. Each well then received 100 µL of zoospore suspension at a density of ~1500 zoospores per mL, resulting in a final volume of 200 µL 2% cV8 with 100 µM test compound and ~150 zoospores per well. Control wells were as above but were amended with 1% DMSO instead of the test compound. Plates were incubated for 24 h at 25 °C in darkness and qualitatively assessed for zoospore germination and mycelial growth using a Nikon SMZ-745T dissecting microscope.

Compounds that prevented zoospore germination relative to the negative control wells were then assayed for inhibition of mycelial growth at a range of concentrations to determine IC_50_ values. The mycelial growth IC_50_ assays were carried out in 24-well plates, with each well containing 1 mL cornmeal agar amended with the test compound at one of six concentrations (concentration ranges varied among compounds based on preliminary assay results (data not shown)). Negative control wells contained 1 mL cornmeal agar amended with DMSO at a final concentration of 1%. Plugs (~2 mm) were removed from the edge of actively growing mycelial mats and added to the centre of each well, and plates were then incubated for ~24 h at 25 °C in the dark. Mycelial mat diameters were measured (two perpendicular measurements were averaged for each well) and growth inhibition was calculated by subtracting the treatment mat diameter from the negative control mat diameter. To calculate the IC_50_ values, compound concentrations were log-transformed, and non-linear regression with curve fitting (by least squares) was carried out using GraphPad Prism version 6.0. 

## Figures and Tables

**Table 1:** Half-maximal mycelial inhibition concentrations (IC_50_s) of compounds active against *P. cinnamomi* and *P. agathidicida*.

Compound ID	*P. cinnamomi* IC_50_ µM	*P. agathidicida* IC_50_ µM
1	ND	92 (64–190) *
2	6.9 (5.6–8.4)	5.5 (3.7–8.0)
3	35 (27–46)	24 (13–47)
4	61 (42–91)	20 (16–24)
5	51 (46–56)	56 (32–210)
6	50 (29–84)	ND
7	82 (57–130)	ND
8	38 (31–46)	19 (16–24)
9	ND	23 (16–32)
10	67 (59–77)	76 (50–190)
11	72 (63–82)	100 (80–130)
12	43 (36–49)	42 (29–93)
13	22 (19–26)	26 (11–140)
14	35 (32–38)	24 (20–29) *
15	9.2 (8.3–10)	6.4 (5.3–7.6)
16	ND	220 (130–940) *
17	37 (29–48)	71 (38–460)
18	34 (26–44)	ND
19	26 (22–30)	36 (27–49)
20	3.4 (1.3–5.4)	3.4 (1.7–5.3)
21	11 (5.0–27)	9.6 (6.6–14)

Values in parentheses are 95% confidence intervals. Three replicates were performed, except when here noted; * indicates assays done in duplicate due to insufficient amount of compound. ND: No data (compound was not inhibitory in initial screening).

## Data Availability

Data is contained within the article or Supplementary Materials.

## Supplementary Materials

The following are available online, Table S1. Plate 1–4; Tables S2 and S3. Half-maximal mycelial inhibition concentrations (IC50s).

## Sample Availability

Samples of the compounds are not available from the authors.
