# Supplementary material for: Screening a Natural Product-Inspired Library for Anti-Phytophthora Activities
Source: Molecules. 2021 Mar 24;26(7):1819. doi: 10.3390/molecules26071819 (PMC8037946; doi:10.3390/molecules26071819)
Supplement: Supplementary file 1 [file molecules-26-01819-s001.zip › Supp Tables 2 and 3.pdf]

Supplementary Information

Screening a natural product-inspired library for anti-*Phytophthora* activities.

Scott A. Lawrence [1], Hannah F. Robinson [2], Daniel P. Furkert [3], Margaret A. Brimble [3] and Monica L. Gerth [2,*]

[1] Department of Microbiology and Immunology, University of Otago, Dunedin, New Zealand
[2] School of Biological Sciences, Victoria University of Wellington, Wellington, New Zealand
[3] School of Chemical Sciences, University of Auckland, Auckland, New Zealand

* Correspondence: monica.gerth@vuw.ac.nz

Supplementary Table S2. Half-maximal mycelial inhibition concentrations (IC_{50}s) of compounds structurally similar to hits **11** and **12**. IC_{50} values are reported in μM, and values in parentheses are 95% confidence intervals (n=3).

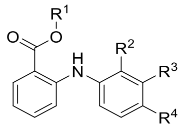

Brimble Library Reference #	R^1	R^2	R^3	R^4	*P. cinnamomi* IC_{50} (μM)	*P. agathidicida* IC_{50} (μM)
**12**	**CH_3**	**H**	**OH**	**H**	**72 (63 – 82)**	**30 (23 – 38)**
**13**	**CH_3**	**H**	**H**	**OH**	**42 (36 – 49)**	**42 (29-93)**
1833	CH_3	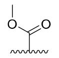	H	OH	95 (86 - 110)	130 (100- 160)
1834	CH_3	H	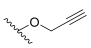	H	No inhibition	190 (170 - 200)
1836	CH_3	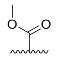	H	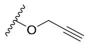	No inhibition	150 (130 - 160)
1837	CH_3	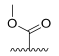	H	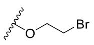	No inhibition	100 (97 - 110)
1842	CH_3	H	H	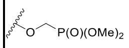	No inhibition	160 (150 - 180)
1843	CH_3	H	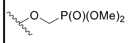	H	No inhibition	190 (190 - 200)
1844	H	H	H	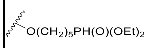	No inhibition	170 (160 - 190)
1845	CH_3	H	H	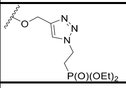	380 (180 - 2800)	140 (120 - 160)
1846	H	H	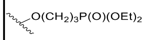	H	340 (35 – 3300)	120 (96 - 150)
1848	H	H	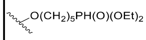	H	No inhibition	150 (120 - 180)

Supplementary Table S3. Half-maximal mycelial inhibition concentrations (IC_{50}s) of compounds structurally similar to **15.** IC_{50} values are reported in µM, and values in parentheses are 95% confidence intervals (n=3).

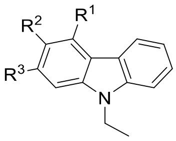

Brimble Library Reference #	R^1	R^2	R^3	*P. cinnamomi* IC_{50} (µM)	*P. agathidicida* IC_{50} (µM)
**16**	**H**	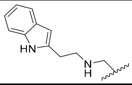	**H**	**9.2 (8.3 – 10)**	**6.4 (5.3 – 7.6)**
47	H	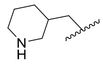	H	32 (25 – 40)	28 (25 - 31)
48	H	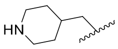	H	46 (40 - 52)	27 (26 - 29)
49	H	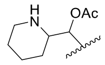	H	No inhibition	69 (58 - 83)
50	H	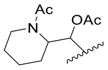	H	81 (66 - 110)	66 (56 - 78)
51	H	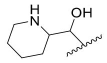	H	20 (17 - 24)	30 (22 - 40)
52	H	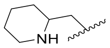	H	68 (58 -78)	54 (48 - 59)
54	H	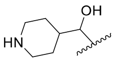	H	160 (130 - 230)	95 (76 - 120)
55	H	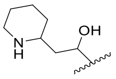	H	75 (65 - 89)	86 (77 - 97)
56	H	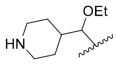	H	49 (40 - 58)	46 (36 - 58)
57	H	H	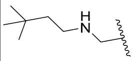	29 (26 - 32)	18 (16 - 19)
63	H	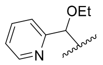	H	No inhibition	150 (130 - 170)
64	H	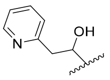	H	46 (34 - 68)	202 (190 - 220)
65	H	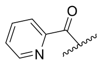	H	No inhibition	160 (140 - 180)
66	CH_3	H	H	29 (22 - 37)	120 (110 - 130)
